# Retrospective evaluation of the use of the International Myeloma Working Group response criteria in dogs with secretory multiple myeloma

**DOI:** 10.1111/jvim.15967

**Published:** 2020-11-20

**Authors:** A Russell Moore, Adam Harris, Christina Jeffries, Paul R. Avery, Kate Vickery

**Affiliations:** ^1^ Department of Microbiology, Immunology and Pathology Colorado State University Ft. Collins Colorado USA; ^2^ Department of Clinical Sciences Colorado State University Ft. Collins Colorado USA

**Keywords:** canine, electrophoresis, immunofixation, M‐protein

## Abstract

**Background:**

Current recommendations for monitoring disease progression and response to treatment in humans with multiple myeloma include evaluation of serum paraprotein (M‐protein) concentration. Densitometry, species‐specific radial immunodiffusion (RID) and ELISA methods can be used to quantify M‐proteins.

**Objective:**

Retrospectively evaluate use of the International Myeloma Working Group (IMWG) response criteria for humans in dogs with multiple myeloma.

**Animals:**

Sixteen dogs with a diagnosis of multiple myeloma, M‐protein documented by serum protein electrophoresis (SPE) and immunofixation (IF) in an initial sample and subsequent electrophoretic evaluation of serial samples.

**Methods:**

Retrospectively, densitometric M‐proteins, RID and globulins were measured and characterized according to IMWG criteria. Available clinical history was reviewed. Overall survival time (OST) was calculated from initial electrophoretic evaluation to death or last contact.

**Results:**

All cases received some form of nonstandardized chemotherapy. Complete response (CR), a lack of detectable M‐protein by SPE and IF, was documented in 1 case. Median survival was longer for dogs that attained ≥90% densitometric M‐protein reduction (630 days) than for those that did not attain at least 50% reduction in densitometric M‐protein (284 days; log rank *P* = .006). Five dogs were defined as having progressive disease (M‐protein increase of >25% and at least 0.5 g/dL from nadir), which correlated with concurrent or subsequent clinical deterioration. Response criteria categorized by serum globulins or RID was not correlated with OST or clinical findings.

**Conclusions and Clinical Importance:**

Densitometric M‐protein characterized using IMWG response criteria correlated with OST and clinical findings. Densitometric M‐protein detection should be used to monitor dogs with multiple myeloma.

**Abbreviations**
AGE: agarose gel electrophoresis
CR: complete response
Glob: chemically‐derived globulin
IF: immunofixation
Ig: immunoglobulin
MR: minimal response
PD: progressive disease
PR: partial response
SD: stable disease
SPE: serum protein electrophoresis
VGPR: very good partial response

## 1 INTRODUCTION

Multiple myeloma (MM) is a plasma cell‐derived neoplasm which affects both humans (1%‐2% of all tumors) and dogs (approximately 1% of all tumors).^1^ Studies have shown that disease presentation, distribution and progression in the dog mirrors that of affected humans.^2^ Multiple myeloma classically has been diagnosed by documenting ≥2 criteria, including neoplastic plasma cells in the bone marrow or tissue, lytic bone lesions and presence of clonal immunoglobulin paraproteins produced by the neoplastic cells (M‐proteins) that can be either complete immunoglobulins or free light chains (fLC). These clonal immunoglobulins can be found either in serum (traditionally called a monoclonal gammopathy) or urine as fLC (Bence‐Jones proteinuria).^3^, ^4^ Recent work in human medicine using novel therapeutic agents has substantially improved ability to treat MM.^5^ As a result of better disease control, improved and more stringent metrics have been developed to use as biomarkers of disease response to treatment and have been termed response criteria. Uniform response criteria allow categorization of response based on monitoring changes in serum or urine M‐protein concentration, affected fLC (ie, normalization of the κ/λ ratio), and tumor size.^5^ In dogs with MM, universal response criteria have yet to be established. The current commonly used biomarkers, serum chemically‐derived globulin (Glob) quantification, immunoglobulin class‐specific radial immunodiffusion (RID) or ELISA or both have been shown to be inconsistent and unreliable at times.^3^, ^5^, ^6^ Reliable criteria are needed to improve response assessment, which in turn will improve treatment planning and potentially overall outcome. We evaluated application of the International Myeloma Working Group (IMWG) consensus response criteria for serum paraprotein monitoring in humans using densitometric M‐protein and previously used markers, RID or Glob, in dogs with secretory multiple myeloma.

## 2 MATERIALS AND METHODS

### 2.1 Study population

The use of dog samples complied with institutional policies and owner consent. Samples were not solicited for this study. Laboratory identification numbers were used to ensure proper identification.

The archives of the Colorado State University (CSU) Clinical Pathology Laboratory were searched for cases with a clinical diagnosis of MM, an initial serum sample submitted between January 1, 2016 and January 1, 2020, a diagnosis of monoclonal or biclonal gammopathy made using diagnostic serum protein electrophoresis (SPE) and immunofixation (IF), and at least 1 subsequent sample evaluated by electrophoresis. Samples submitted to the Clinical Pathology Laboratory originated from either the Colorado State University‐Veterinary Teaching Hospital (CSU‐VTH) or outside referral hospitals and had been archived at −80°C. All evaluations were performed at the time of submission or within 4 years; M‐protein concentration has been shown to be stable for this duration.^3^ During this period, dogs with a new diagnosis of monoclonal or biclonal gammopathy seen at CSU‐VTH were noted, and the laboratory information management system was programed to prompt archival of subsequently submitted serum from these dogs. Six cases originated directly from the CSU‐VTH dog population and, for these cases client consent for sample collection was obtained, according to university policy, before collection. Ten cases were submitted from outside referral hospitals directly to the CSU Clinical Pathology Laboratory.

### 2.2 M‐protein characterization and quantification

Total protein concentration was determined using the biuret method (Cobas c501; Roche Diagnostics, Indianapolis, Indiana). Serum protein electrophoresis was performed using previously described methods on a Sebia Hydrasys system (Sebia, France) using Hydragel Protein (E) gels.^3^ Confirmation of involved immunoglobulin class was made by routine IF for IgG‐FC, IgG4, IgA, IgM and light chain using canine‐specific reagents, as previously described.^3^, ^7^ When needed, evaluation for serum fLC was performed using a human‐targeted fLC IF antibody set (Sebia Free light chains, Sebia, France) and the presence of fLC was confirmed using urine protein electrophoresis. The M‐proteins were quantified densitometrically using the corrected perpendicular drop method from SPE.^3^ When M‐protein was not discernable on serial samples by SPE alone, repeat IF was performed.

### 2.3 Radial immunodiffusion

Radial immunodiffusion (RID) and serum Glob determination were performed retrospectively if these measurands were not assessed at the time of initial submission and sample volume was sufficient. Radial immunodiffusion for the involved immunoglobulin heavy chain was performed using commercially available canine immunoglobulin class‐specific RID kits for IgG, IgA, or IgM (Triple J Farms; Kent Laboratories, Bellingham, Washington), following the manufacturer's instructions. Samples without an involved heavy chain were not evaluated by RID.

### 2.4 Globulin

Chemcially‐derived globulin (Glob) concentration was determined as the difference between total protein and serum chemically‐derived albumin concentration determined using the bromocresol green method (Cobas c501; Roche Diagnostics, Indianapolis, Indiana).

### 2.5 Characterization of response

Changes in M‐protein, RID and Glob concentration were calculated and response category assigned using serum‐specific IMWG response criteria, as outlined in Table 1.^5^ Available clinical histories were reviewed by a board‐certified oncologist (KV) and pertinent data including breed, age, date of diagnosis, disease distribution at diagnosis, treatment type and start date, date of progression, and date of death were extracted. Evidence of clinical deterioration was based on owner reports, attending veterinarian impressions and available radiographic, cytologic, and histologic data, as recorded in the clinical history. When a full medical record was not available, information from the sample submission form was used. Overall survival time (OST) was calculated as the time from initial electrophoretic evaluation by CSU Clinical Pathology Laboratory (ie, date of initial SPE at CSU) to death or last known contact. Survival curves were composed with response categories partitioned into 3 groups (progressive disease‐minimal response [PD‐MR], partial response [PR], very good partial response‐complete response [VGPR‐CR]) and evaluated using a log‐rank test then pairwise testing was performed and a Bonferroni‐corrected *P* ≤ .02 was used to test for significance.

**TABLE 1:** Serum‐based IMWG consensus response criteria applied to canine secretory multiple myeloma

Category	Serum‐based criteria
CR—complete response	M‐protein: Lack of M‐protein by electrophoresis and immunofixation RID or Globulin: Within reference interval
VGPR—very good partial response	M‐protein: ≥90% reduction by electrophoresis but M‐protein present by immunofixation RID or Globulin: ≥90% reduction and above upper reference limit
PR—partial response	All measurands: ≥50% reduction
MR—minimal response	All measurands: ≥25% reduction. Absolute decrease ≥0.5 g/dL
SD—stable disease	All measurands: <25% reduction or increase
PD—progressive disease	All measurands: ≥25% increase from lowest concentration and – If lowest concentration <5/dL, absolute increase ≥0.5 g/dL – If lowest concentration ≥5 g/dL, absolute increase ≥1.0 g/dL

*Note:* Change is relative to initial value unless otherwise stated.

Statistical analysis was performed using Excel (Microsoft Office 2016; Microsoft, Microsoft, Redmond, Washington), with Real Statistics Resource Pack software (Release 5.4, www.real-statistics.com). Additional statistical analysis was performed using GraphPad Prism 8 (GraphPad Software, Inc, La Jolla, California).

## 3 RESULTS

Sixteen cases of secretory multiple myeloma in dogs met inclusion criteria. Attending veterinarian records were available in 13/16 cases. Demographics, disease distribution and treatment protocols are found in the Supporting Information. The monoclonal proteins all were found in serum and were classified as 3 IgG, 11 IgA, 1 IgM, and 1 fLC only (Supporting Information).

Archived serum or SPE results were available from 71 samples. Median number of samples per case was 3. Maximum number of samples from any case was 11. A pretreatment sample was available in 11/16 cases; the remaining 5 initial samples were collected during treatment and were used when assigning response category but were not considered when describing total protein or Glob concentrations of pretreatment or follow‐up samples. Serum protein electrophoresis, including assessment of total protein concentration, was performed on all 71 samples and serum Glob concentration was assessed in 67 samples. Radial immunodiffusion for the involved Ig class was available for 66 samples. Descriptive statistics for all samples and the pretreatment and follow‐up samples are included in Table 2. Graphs of 5 representative cases showing progression of results and comparison of response criteria category for each measurand during treatment are presented in the Supporting Information.

**TABLE 2:** Descriptive statistics of serum samples from 16 dogs with secretory multiple myeloma

	All classes	IgG	IgA	IgM	fLC
Number of cases	16	3	11	1	1
Number of samples	71	12	54	3	2
Pretreatment samples					
Number	11	3	6	1	1
Total protein					
Average (min‐max) g/dL	10.6 (5.9‐14.6)	10.9 (7.6‐14.2)	9.1 (14.2‐6.6)	9.8	5.9
Number within RI	2	0	1	0	1
Densitometric M‐protein					
Average (min‐max) g/dL	5.66 (0.77‐10.39)	4.66 (1.35‐8.85)	7.08 (1.87‐10.39)	4.99	0.77
Class‐specific RID					
Average (min‐max) g/dL	13.368 (0.119‐36.122)	7.548 (5.456‐11.221)	18.487 (6.019‐36.122)	0.119	NA
Number within RI	1	0	0	1	NA
Globulin					
Globulin measured	67	11	51	3	2
Average (min‐max) g/dL	8.1 (3.4‐12.8)	8.0 (4.1‐12.7)	9.2 (3.4–12.8)	7.6	2.6
Number within RI	1	0	0	0	1
Follow‐up samples					
Number	55	9	43	2	1
Total protein					
Number within RI	39	3	33	2	1
Densitometric M‐protein					
Quantifiable by SPE	50	9	40	0	1
Detected but not quantifiable	4	0	3	1	0
Not detected by SPE or IF	1	0	0	1	0
M‐protein detectible by SPE or IF & Total protein within RI	38	3	33	1	1
RID					
RID measured	51	8	41	2	NA
Number within RI	6	0	5	1	NA
M‐protein detectible by SPE or IF & RID within RI	6	0	5	1	NA
Globulin					
Globulin measured	51	8	40	2	1
Number within RI	28	2	23	2	1
M‐protein detectible by SPE or IF & globulin within RI	27	2	23	1	1

*Note:* Samples were evaluated together (all cases) and stratified by involved immunoglobulin class.

Abbreviations: fLC, free light chain; RI, reference interval.

The relationship of M‐protein with RID or serum Glob concentration results was similar to previously published findings,^3^ with serum Glob concentration showing a positive bias over densitometric M‐protein, with IgA RID showing positive constant bias and proportional bias, IgG showing constant bias at low densitometric M‐protein concentrations and IgM RID not being >2 times the upper limit, independent of densitometric IgM M‐protein concentration (Supporting Information Figure 1).

### 3.1 Densitometric M‐protein

The initial sample from all cases had quantifiable M‐protein by densitometry; all cases were included in evaluation of OST as determined by densitometric M‐protein concentration. Statistical significance was found when cases were partitioned into groups of PD‐MR (<50% densitometric M‐protein reduction), PR (50%‐90% densitometric reduction) and VGPR‐CR (>90% densitometric M‐protein reduction), with median survival 284, 496, and 630 days, respectively (log rank *P* = .007; Table 3 and Figure 2A). Pairwise evaluation identified longer survival with ≥90% reduction (VGPR‐CR) than with <50% reduction (PD‐MR) in densitometric M‐protein (log rank *P* = .006).

**TABLE 3:** Response category and outcome data from 16 dogs with secretory multiple myeloma

	M‐protein	RID	Globulin
# Cases evaluated	16	14	13
Best response			
CR	1	4	7
VGPR	6	2	0
PR	4	4	1
MR	1	1	3
SD	3	0	2
PD	1	3	0
Median survival (days)			
PD‐MR	284	468.5	408
PR	496	325	463
VGPR‐CR	630	506	630
Three‐group Log‐rank *P*‐value	.01	.57	.60
PD‐MR vrs PR *P*‐value	.05	.53	.95
PD‐MR vrs VGPR‐CR *P*‐value	.01	.82	.40
PR vrs VGPR‐CR *P*‐value	.32	.23	.45

*Note:* Data were evaluated using 3 different methods to assess paraprotein concentration, densitometric M‐protein, involved class‐specific RID and chemically‐derived globulin. Survival for each measurand and result of Log‐rank evaluation of the cases partitioned in 3 groups and pairwise comparisons are provided.

Abbreviations: CR, complete response; VGPR, very good partial response; PR, partial response; MR, minimal response; SD, stable disease; PD, progressive disease.

Densitometric M‐protein concentration defined progressive disease (PD) in 10 samples from 5 cases, all of which had concurrent or subsequent clinical deterioration. Progressive disease was found in case 6, concurrent with incomplete resection of a presumed solitary plasma cell tumor; subsequent initiation of chemotherapy was concurrent with categorization of minimal response (MR). All dogs that experienced clinical deterioration had concurrent increases in densitometric M‐protein concentration.

### 3.2 Radial immunodiffusion

**FIGURE 1:**
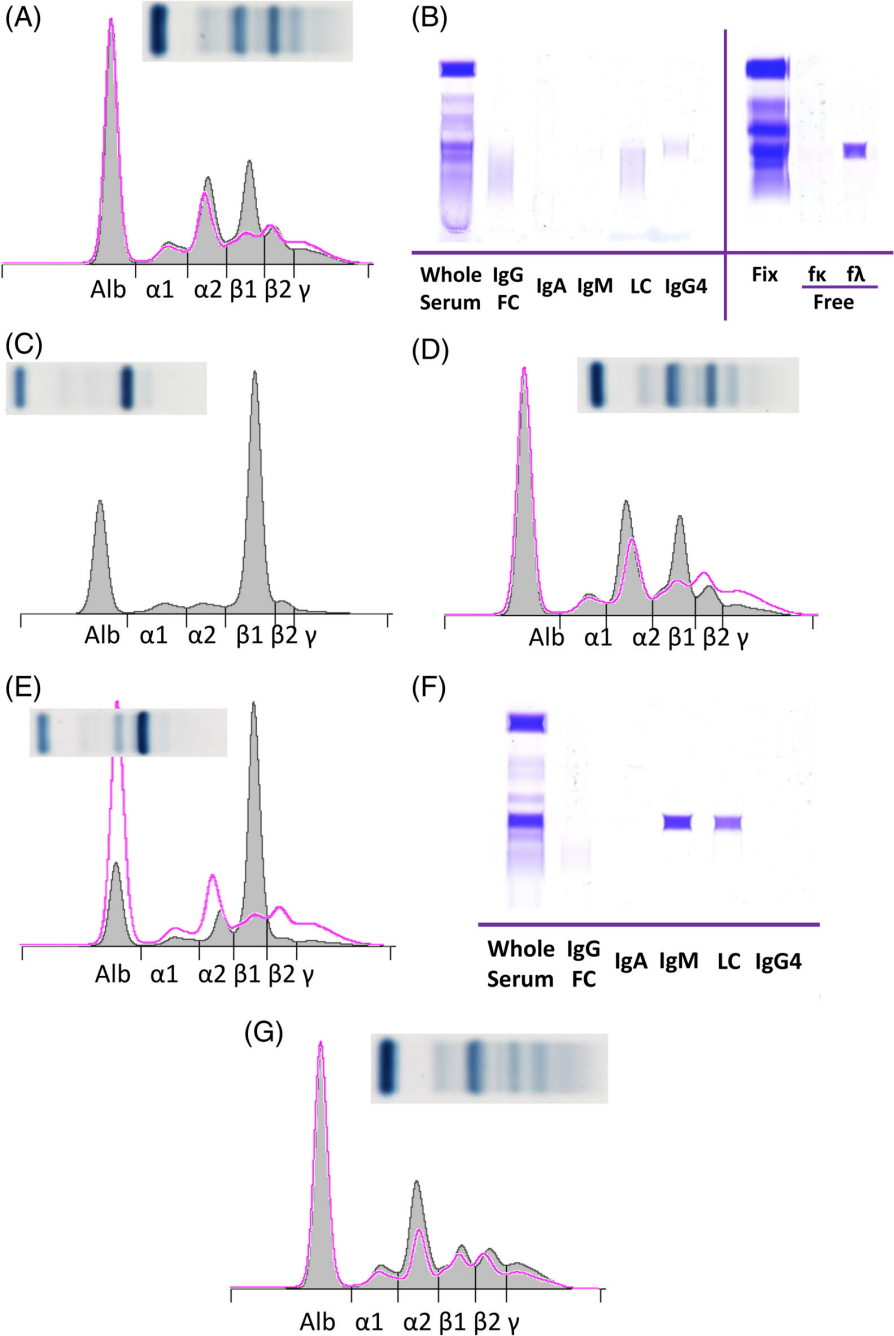
Serum protein electrophoresis and immunofixation from 2 dogs with multiple myeloma and a serum β‐1 M‐protein. A‐D, Pretreatment serum and midtreatment serum and urine from an 8‐year‐old female spayed Golden Retriever, case 16. A, There is a slightly more prominent β‐1 restricted band compared to the control sample (pink tracing), suggestive but not definitive for an M‐protein. B, The restricted band labels only with the anti‐human free λ (fλ) reagent on immunofixation of the serum, consistent with a serum free light chain M‐protein. C, A similar restricted band was present in the urine, diagnostic of Bence‐Jones proteinuria. D, The previously documented M‐protein peak remained unchanged during treatment. E‐G, Pretreatment and midtreatment serum samples from a 12‐year‐old male castrated American Staffordshire Terrier, case 15. E, There is a tall narrow β‐1 restricted band and relative hypoalbuminemia relative to the control sample (pink tracing). F, The restricted band labels with both IgM and light chain, diagnostic of a complete IgM M‐protein. G, A serum sample during treatment demonstrates resolution of the M‐protein. The M‐protein was not present on immunofixation, indicating the dog was in complete remission. Image A, C, D, E, and G image inset is an image of the electrophoretic gel. ALB, albumin; LC, light chain; fκ, anti‐human free κ; fλ, anti‐human free λ

**FIGURE 2:**
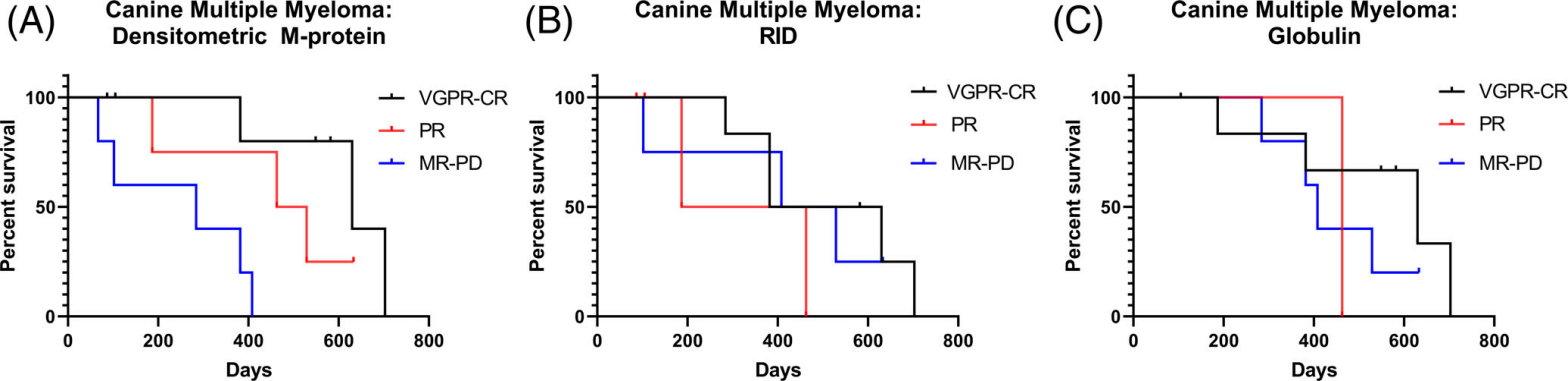
Kaplan‐Meier curves for overall survival time for canine cases of secretory multiple myeloma categorized using the IMWG response criteria based on, A, densitometric M‐protein, B, immunoglobulin‐class‐specific radial immunodiffusion, or C, chemically‐derived globulin. CR, complete response; MR, minimal response; PD = progressive disease; PR, partial response; VGPR, very good partial response

All 3 samples from the IgM gammopathy case had IgM RID performed, but the measured amount of IgM did not correlate with the M‐protein quantification, as previously determined. The initial sample with a 4.99 g/dL densitometric M‐protein concentration (Figure 1E‐G) had an IgM RID result within reference limits (119 mg/dL; reference interval [RI], 100‐200 mg/dL).^3^ This finding was interpreted to indicate that RID did not detect IgM paraproteins in this case. Response assessment based on RID could not be performed in the fLC and IgM cases because the gammopathy could not be reliably detected in the initial samples. Response assessment based on RID was evaluated in all IgA and IgG cases. No difference was found when cases were evaluated as 3 groups (PD‐MR, PR, VGPR‐CR) with median survival of 468, 325 and 506 days, respectively (log rank *P* = .57; Table 3 and Figure 2B). Pairwise comparison failed to identify a difference in survival (log rank *P* = .23‐.83).

Change in RID results was categorized as evidence of PD in 18 samples from 9 cases. This finding coincided with evidence of clinical deterioration of the dog in 11 samples. The sample was collected before evidence of clinical deterioration by 36, 72, or 278 days in 3 samples, and was the last sample collected before death by 145 days in 1 sample from a case with incomplete history. Progressive disease defined by RID was not associated with evidence of clinical deterioration in 4 instances, all of which were categorized as having some form of response by densitometry. Progressive disease defined by RID correlated with densitometric M‐protein‐defined PD in 9/18 samples. Change in RID was categorized as evidence of CR in 6 samples from 4 cases. In 5 samples, RID‐defined CR coincided with clinical improvement, but M‐protein still was identified by SPE and IF. Case 6 had RID‐defined CR concurrent with densitometric‐defined PD and evidence of systemic involvement associated with a previously excised solitary plasma cell tumor.

Very poor correlation was found between densitometric M‐protein‐ and RID‐based IMWG category (weighted kappa, 0.26; 95% confidence interval [CI], 0.09‐0.44).

### 3.3 Globulin

Serum Glob concentration failed to detect the gammopathy in all samples from 3 cases (the fLC case and 2 IgA cases that initially were sampled after beginning treatment). These cases were not used for evaluation of serum Glob concentration to characterize disease progression. Percentage change was calculated based on serum Glob concentration in 13 cases, with at least 1 serum Glob concentration above the upper reference limit. No difference in survival was documented when cases were evaluated in 3 groups (PD‐MR, PR, VGPR‐CR). Median survival was 463, 408 and 630 days, respectively (log rank *P* = .60; Table 3 and Figure 2C). Pairwise evaluation failed to document a difference in survival (log rank *P* = .40‐.95).

Change in serum Glob concentration was categorized as PD in 12 samples from 5 cases. This change coincided with evidence of clinical deterioration in 9 samples and preceded evidence of clinical deterioration by 72, 93 and 134 days in 3 samples from 2 cases. Serum Glob concentration was within reference limits (interpreted as CR), and no evidence of clinical deterioration was found in 28 samples, but M‐protein was detected by electrophoretic techniques in 27 of these 28 samples.

Very poor correlation was found for M‐protein‐based IMWG response category between densitometric M‐protein and serum Glob concentration‐based category (weighted kappa, 0.38; 95% CI, 0.25‐0.52).

## 4 DISCUSSION

We used a validated M‐protein measurement to evaluate progression of MM in dogs. Our results indicate that use of the serum densitometric M‐protein is a feasible monitoring tool compared to RID or serum Glob concentration and that M‐protein‐based IMWG criteria assessment was associated with survival in dogs with secretory MM. Furthermore, M‐protein‐based assessment could not be used interchangeably with class‐specific RID or serum Glob concentration to assign cases to IMWG response category and better aligned with clinical findings than class‐specific RID or changes in serum Glob concentration.

Traditionally, SPE has been used to confirm the diagnosis of monoclonal or biclonal gammopathy, but not to monitor progression of disease in affected dogs. Densitometric quantitation of M‐protein has been used in human medicine for many years, but only recently has been validated for use in dogs.^3^ Electrophoretic fractions, and not specific measurement of M‐protein, have been used previously to monitor cases of MM in dogs.^1^, ^8^, ^9^, ^10^ In contrast to reports in the human medical literature, where non‐γ‐globlulin M‐protein quantification is more inaccurate and imprecise, evaluation in dogs suggests that location of the M‐protein within the electrophoretic profile does not affect analytical accuracy for quantification of low concentrations of M‐proteins.^3^, ^11^ Most of the quantified M‐protein bands in our study were in the β‐globulin region and clinical relevance still was found, supporting the assertion that electrophoretic location of the M‐protein does not affect the clinical accuracy of M‐protein quantification in dogs.

Immunofixation combined with SPE increases the sensitivity for detection of monoclonal and biclonal gammopathies.^12^, ^13^ Using antibodies against canine proteins, it also allows characterization of the involved heavy chain and associated light chains but does not distinguish light chain class.^14^ Until recently, involved heavy chain class was not thought to be associated with survival or disease progression in humans, but heavy chain class still was determined for monitoring purposes because some treatments can result in novel, oligoclonal gammopathies that are not evidence of worsening disease.^15^, ^16^ A recent study in humans suggested that IgA gammopathies had poorer prognosis.^17^ Our study dataset contained very few non‐IgA cases, making evaluation of this correlation difficult but a previous study in dogs did not detect an association between heavy chain class and prognosis.^1^ A larger dataset is needed, but there does not appear to be a survival advantage based on the involved heavy chain class.

The multidrug‐resistant urinary tract infection in the fLC‐only MM case may be a confounding factor because it likely affected renal function and contributed to the dog's decline. This dog had a serum M‐protein concentration of 0.77 g/dL at initial diagnosis. This concentration is well above the 0.08 g/dL concentration shown to be associated with a poor prognosis and a higher risk of renal insufficiency in humans, resulting from the nephrotoxic effects of fLC in serum.^233^ This nephrotoxicity can be reversed by decreasing the serum fLC concentration using antimyeloma treatments.^11^, ^18^ When chemotherapy was administered in the fLC‐only MM case, serum M‐protein concentration showed no evidence of response, renal function worsened and euthanasia was elected 67 days after diagnosis. Although the bacterial infection may have contributed to the decline in renal function, an association of Bence‐Jones proteinuria and poorer prognosis in dogs with MM that produced an intact immunoglobulin has been reported.^1^ Our data suggest that sustained increased serum fLC concentration may be a poor prognostic factor in affected dogs. Further evaluation of the effect of increased serum fLC concentration on prognosis in affected dogs is warranted.

Few cases consistent with fLC‐only MM in dogs have been reported,^8^, ^18^ which may reflect previous challenges in recognizing and diagnosing fLC‐only MM. Free light chains are freely filtered from the serum into urine. Thus, high serum fLC concentrations are not expected, and affected dogs typically have normal, or near normal, serum total protein, Glob concentrations, and serum electrophoretograms.^8^, ^18^, ^19^ Evaluation of urine protein electrophoresis, fLC IF or both often is needed to identify such cases. In humans, approximately 16% of all myelomas are fLC‐only and, using very sensitive assays, 95% of myelomas are found to be associated with abnormal serum fLC concentrations.^16^ It is likely that increased serum fLC concentrations and fLC‐only MM remain underrecognized in dogs because the need for SPE is not recognized in these dogs, and because an assay for determining serum fLC concentration has not been available previously.

Several reports in dogs have described class‐specific RID to monitor response to treatment.^5^, ^6^ Radial immunodiffusion and other (nephelometric) methods for monitoring paraprotein concentration have been shown to produce results that are method‐specific in dogs and humans.^3^, ^13^, ^20^ In our dataset, 1 sample had an IgA concentration of 36 g/dL identified by RID in a dog with a serum Glob concentration of 12.8 g/dL and another had an IgM concentration of 0.119 g/dL identified by RID in a dog with an IgM M‐protein concentration of 4.99 g/dL. Despite these physiologically impossible RID numerical results, it is still possible that the relative changes in RID results might have been clinically useful. Current recommendations in human medicine suggest use of nephelometric M‐protein quantification when densitometry is unavailable, while citing similar challenges with supraphysiologic results.^11^ Radial immunodiffusion predicted disease progression in 2 cases earlier than did densitometric M‐protein results, which suggests it may be more sensitive. However, it also indicated disease progression midtreatment and at other times when disease progression could not be confirmed in 4 other cases. Ultimately, RID‐based response criteria assessment was not associated with OST. Our study suggests that RID is not clinically useful for monitoring immunoglobulin paraproteins.

Serum total protein and Glob concentrations often are used both to identify and monitor monoclonal or biclonal gammopathy. However, these measurands can be insensitive for detecting and monitoring gammopathies because dogs can be normoproteinemic and normoglobuinemic or even hypoglobulinemic and still have a gammopathy.^13^, ^21^ In our study, 2 pretreatment samples had normal serum total protein concentration and 1 pretreatment sample had normal Glob concentration, but all pretreatment samples had documented M‐proteins. Additionally, M‐proteins were documented in most follow‐up samples, with normal serum total protein or Glob concentrations. Lymphoproliferative disease and high concentration M‐protein can induce compensatory suppression of normal immunoglobulins, which then rebound with resolution of the disease. Common processes that affect serum Glob concentration, such as an acute phase protein response, then can occur concurrently with a monoclonal or biclonal gammopathy.^20^ These effects can combine to mask the specific changes in paraprotein. Serum Glob concentration was insensitive to changes in M‐protein, missing 27 samples with M‐protein present, and was not associated with clinical deterioration. Normal serum total protein and Glob concentration should not be used to rule out the presence of M‐protein and a gammopathy.

The retrospective nature of our study limited our ability to answer some relevant and important questions about the clinical utility of M‐protein monitoring. The true percentage of cases that achieved CR and other categories of treatment response cannot be determined from our dataset. One case had densitometric M‐protein‐defined CR, but lack of documented CR may not accurately reflect treatment response in all cases. Other cases in our dataset may have had better resolution of their paraproteinemia than was identified, but samples may not have been collected at that time point because clinical need for sampling was not perceived. This situation may have confounded evaluation of the benefits of attaining more decrease in M‐protein concentration. Treatment was not standardized in our study, further limiting our ability to determine optimal response rates and appropriate reevaluation time points. The ability of changes in M‐protein concentration to predict changes in clinical condition cannot be fully evaluated because the sampling period was not consistent across all cases, but instead was a function of when the attending veterinarian judged that repeat evaluation was warranted. However, longer median survival was documented in cases that attained ≥90% decrease in densitometric M‐protein. Additionally, median survival time was longer in dogs that attained between 50% and 90% decrease than in those that attained a <50% decrease in densitometric M‐protein and shorter than in those that obtained ≥90% decrease, but these pairwise comparisons were not significant. Attaining at least a 90% densitometric M‐protein decrease may be a clinically reasonable target for affected dogs. A prospective study with increased numbers of cases is needed to better characterize use of densitometric M‐protein monitoring in dogs with secretory multiple myeloma.

Our study found that the IMWG consensus response criteria based on serum M‐protein were associated with both specific clinical events and OST whereas categorization based on RID and serum Glob concentration were not. Increased use of the electrophoresis‐based techniques, including evaluation for serum fLC concentration and densitometric M‐protein evaluation as a means of characterizing and monitoring dogs with secretory multiple myeloma is recommended.

## CONFLICT OF INTEREST DECLARATION

Authors declare no conflict of interest.

## OFF‐LABEL ANTIMICROBIAL DECLARATION

Authors declare no off‐label use of antimicrobials.

## INSTITUTIONAL ANIMAL CARE AND USE COMMITTEE (IACUC) OR OTHER APPROVAL DECLARATION

Authors declare use of dog samples was compliant with institutional policies.

## HUMAN ETHICS APPROVAL DECLARATION

Authors declare human ethics approval was not needed for this study.

## Supporting information


**Data S1**: Supporting Information.
