# Supplementary material for: Retrospective evaluation of the use of the International Myeloma Working Group response criteria in dogs with secretory multiple myeloma
Source: J Vet Intern Med. 2020 Nov 20;35(1):442–50. doi: 10.1111/jvim.15967 (PMC7848390; doi:10.1111/jvim.15967)
Supplement: Supplementary file 1 — Data S1: Supporting Information. [file JVIM-35-442-s001.pdf]

Supplemental Figure 1. Correlation of IgA radial immunodiffusion (RID), IgG RID or biochemical globulin with densitometric M-protein in 64 samples from 16 cases.

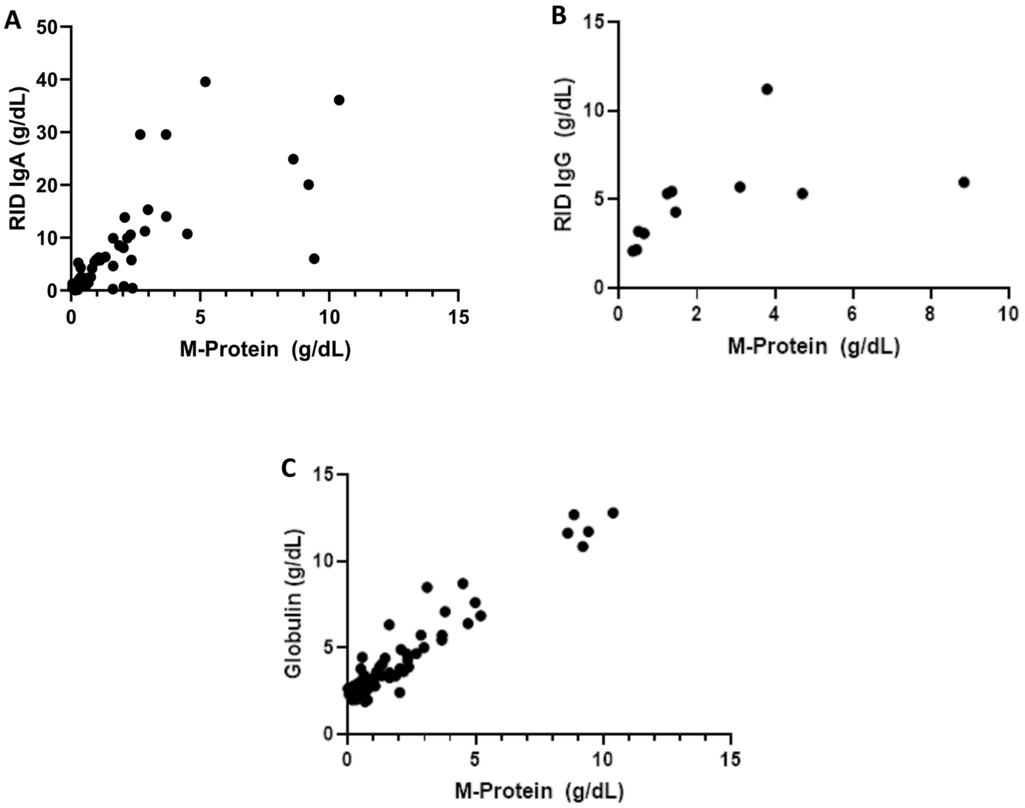

Supplemental Figure 2. Comparison of densitometric M-protein, class specific radial immunodiffusion (RID) and biochemical globulins in serial samples from 5 dogs with secretory multiple myeloma. Data is percentage after normalization to the pre-treatment sample. Results within reference limits are denoted with a ▲. Globulin RI 1.5-3.2 g/dl, IgA RID RI 0.040-0.160 g/dl, IgG RID RI 1.000-2.000 g/dl, IgM RID RI 0.100-0.200 g/dl. Samples concurrent with clinical deterioration are denoted with an open symbol. Initial value and subsequent response category for each measurand and time point are included in the table below the chart.

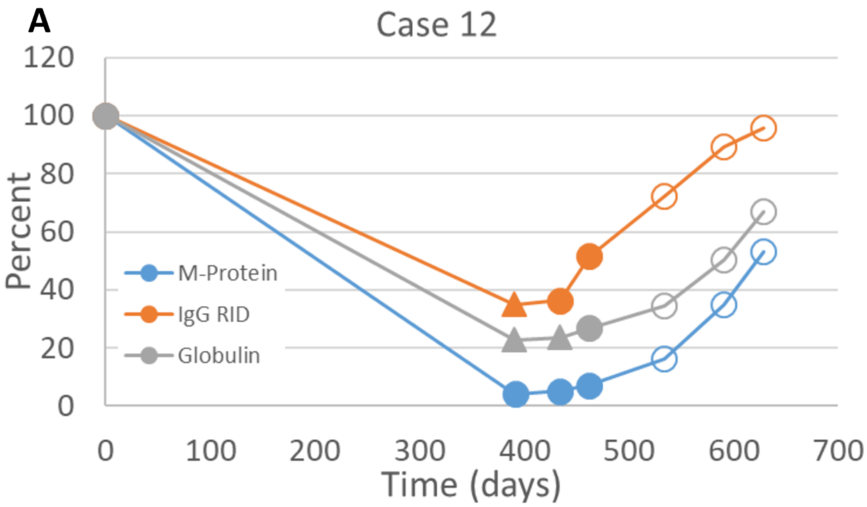

	Initial	Day					
	g/dl	391	434	462	534	591	629
M-protein	8.85	VGPR	VGPR	VGPR	PD	PD	PD
IgG RID	5.966	CR	PR	PD	PD	PD	PD
Globulin	12.7	CR	CR	PD	PD	PD	PD

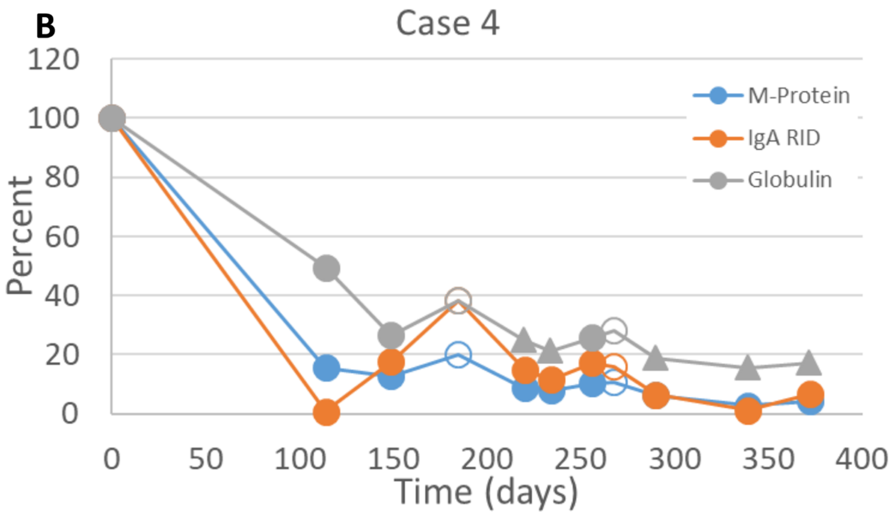

	Initial	Day									
	g/dl	114	149	185	220	234	256	268	290	339	372
M-protein	10.39	PR	PR	PD	VGPR	VGPR	VGPR	PD	VGPR	VGPR	VGPR
IgA RID	36.122	VGPR	PD	PD	PR	PR	PR	PD	VGPR	VGPR	VGPR
Globulin	12.8	PR	PR	PD	CR	CR	PD	PD	CR	CR	CR

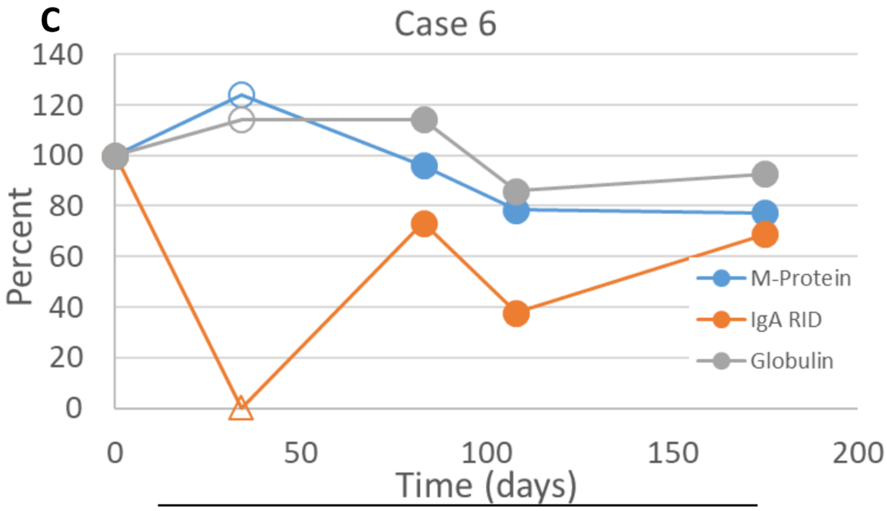

	Initial	Day			
	g/dl	34	83	108	175
M-protein	2.98	PD	SD	MR	MR
IgA RID	15.307	CR	PD	MR	PD
Globulin	5.0	SD	SD	SD	SD

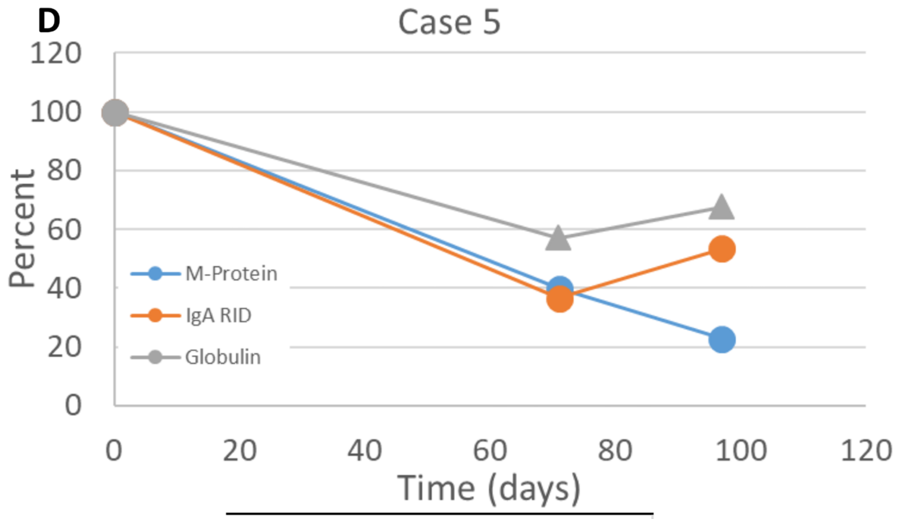

	Initial	Day	
	g/dl	71	97
M-protein	1.36	PR	PR
IgA RID	5.456	PR	PD
Globulin	4.1	CR	CR

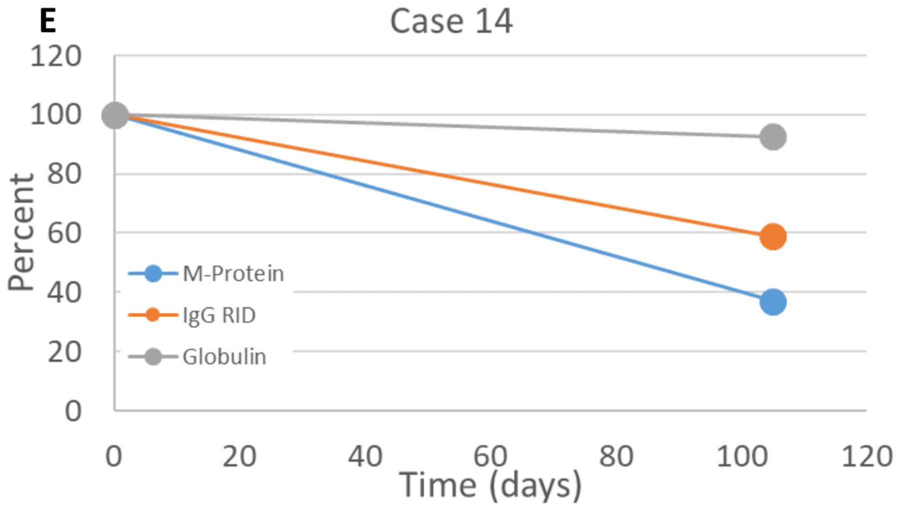

	Initial	Day
	g/dl	105
M-protein	1.35	PR
IgG RID	5.456	MR
Globulin	4.1	SD

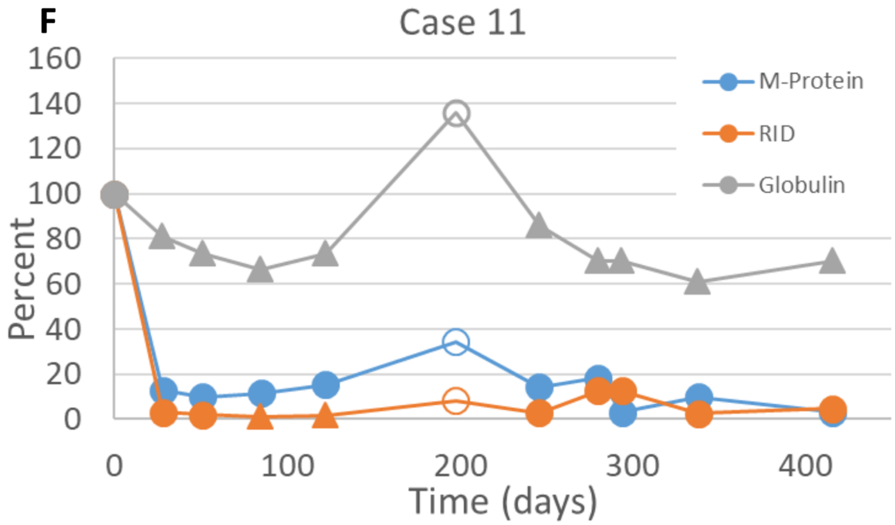

	Initial	Day									
	g/dl	28	51	85	122	198	246	280	294	338	416
M-protein	1.63	PR	VGPR	PR	PR	PD	PR	PR	VGPR	VGPR	VGPR
IgA RID	9.897	VGPR	VGPR	CR	CR	PD	VGPR	PD	SD	PR	PR
Globulin	3.3	CR	CR	CR	CR	PD	CR	CR	CR	CR	CR

Supplemental Table 1: Demographic and clinical information of 16 dogs with secretory multiple myeloma

Case #	Age	Sex	Breed	Clinical Distribution	Treatment	Records Available
1	12	MN	Mix	Unknown	Unknown	N
2	9	MC	Labrador Retriever	T1 vertebral body, Plasma cell tumor in spleen (histology)	Unknown	Y
3	7	MC	Golden Retriever	Peripheral lymphadenopathy Plasma cell tumor of spleen (cytology), Splenomegaly, Pancytopenia w plasmacytosis (peripheral blood smear cytology), Bilateral retinal hemorrhage, Coagulopathy	Tanovea/Prednisone	Y
4	7	FS	Labrador Retriever	Pancytopenia, Hypercalcemia, Azotemia, Multifocal osteolytic bone lesions, Bilateral retinal detachment, Splenomegaly. Plasma cell tumor in spleen (cytology)	Melphalan/Prednisone	Y
5	10	MN	German shepherd mix	Plasmacytosis of spleen, Hypercalcemia, Thrombocytopenia	Melphalan/Prednisone	Y
6	11	FS	Labrador Retriever	Hypercalcemia, Multiple osteolytic bone lesions, Plasma cell tumor 6th rib (histology)	Surgery and Melphalan/Prednisone	Y

Case #	Age	Sex	Breed	Clinical Distribution	Treatment	Records Available
7	10	FS	Labrador Retriever	Plasmacytosis of spleen, Multiple osteolytic bone lesions	Surgery, Palliative RT, Zoledronate, Melphalan/Prednisone	Y
8	11		Spitz cross	Pancytopenia, Hepatosplenomegaly, Abdominal lymphadenopathy, Possible C2 vertebral lysis	Melphalan/Prednisone	Y
9	7	FS	Mixed Breed Dog	Pancytopenia, Hypercalcemia, Azotemia, Coagulopathy, Right Ilium lytic bone lesion, Splenomegaly, Abdominal lymphadenopathy, Plasma cell tumor of spleen (Cytology)	Melphalan/Prednisone	Y
10	8	MC	Min Schnauzer	Multiple osteolytic bone lesions	Cyclophosphamide and Zoledronate	Y
11	9	FS	Labrador Retriever	Plasmacytosis of spleen and liver, Thrombocytopenia, Multiple lytic bone lesions	Tanovea/Prednisone	Y
12	9	FS	Mixed Breed Dog	Pancytopenia, Hypercalcemia, Plasma cell tumor of spleen (cytology)	Melphalan/Prednisone	Y
13		MC	Border Collie	Unknown	Unknown	N

Case #	Age	Sex	Breed	Clinical Distribution	Treatment	Records Available
14	10	MC	Shih Tzu	Multifocal vertebral lesions, largest at T1. Plasma cell tumor of T1 (cytology)	Stereotactic RT and Melphalan/Prednisone	Y
15	12	MC	Am Staff Terrier	Unknown	Unknown	N
16	8	FS	Golden Retriever	Multifocal osteolytic bone lesions (R proximal humerus, 2nd rib), Diffuse nodular changes liver and kidney, plasma cell tumor (cytology)	Melphalan/Prednisone, Palliative RT, Zoledronate	Y

Supplemental Table 2. Serum sample, M-protein characterization and outcome data of 16 dogs with secretory multiple myeloma.

Case #	Pre-treatment sample available	# of samples	Involved Class	Location	Survival Time (days)	Outcome
1	N	2	IgA	β-2, γ	529	Deceased
2	N	6	IgA	β-1, β-2	382	Deceased
3	Y	5	IgA	β-2, γ	703	Deceased
4	Y	12	IgA	β-2, γ	382	Deceased
5	N	3	IgA	β-1, β-2	187	Deceased
6	Y	5	IgA	β-2, γ	284	Deceased
7	Y	4	IgA	β-1, β-2	183	Alive
8	N	2	IgA	β-2, γ	157	Alive
9	Y	3	IgA	β-1, β-2	408	Deceased
10	N	2	IgA	β-1, β-2	102	Deceased
11	N	11	IgA	γ, γ	582	Lost to follow-up
12	Y	7	IgG	γ	630	Deceased
13	Y	3	IgG	γ	463	Deceased
14	Y	2	IgG	γ	633	Alive
15	Y	3	IgM	β-1	549	Alive
16	Y	2	FLC	β-1	67	Deceased
